# Comparative study of a novel proximal femoral bionic nail and three conventional cephalomedullary nails for reverse obliquity intertrochanteric fractures: a finite element analysis

**DOI:** 10.3389/fbioe.2024.1393154

**Published:** 2024-06-13

**Authors:** Yanjiang Yang, Yu Tong, Xiaodong Cheng, Yanbin Zhu, Wei Chen, Yunwei Cui, Qi Zhang, Yingze Zhang

**Affiliations:** ^1^ Trauma Emergency Center, Third Hospital of Hebei Medical University, Shijiazhuang, China; ^2^ Orthopaedic Research Institute of Hebei Province, Shijiazhuang, China; ^3^ Key Laboratory of Biomechanics of Hebei Province, Shijiazhuang, China; ^4^ NHC Key Laboratory of Intelligent Orthopaedic Equipment, Shijiazhuang, China; ^5^ Hebei Orthopaedic Clinical Research Center, Shijiazhuang, China

**Keywords:** biomechanics, cephalomedullary nail, finite element analysis, proximal femoral bionic nail, reverse obliquity intertrochanteric fracture

## Abstract

**Purpose:**

Conventional cephalomedullary nails (CMNs) are commonly employed for internal fixation in the treatment of reverse obliquity intertrochanteric (ROI) fractures. However, the limited effectiveness of conventional CMNs in addressing ROI fractures results in significant implant-related complications. To address challenges associated with internal fixation, a novel Proximal Femoral Bionic Nail (PFBN) has been developed.

**Methods:**

In this study, a finite element model was constructed using a normal femoral specimen, and biomechanical verification was conducted using the GOM non-contact optical strain measurement system. Four intramedullary fixation approaches—PFBN, Proximal Femoral Nail Antirotation InterTan nail (ITN), and Gamma nail (Gamma nail)—were employed to address three variations of ROI fractures (AO/OTA 31-A3). The biomechanical stability of the implant models was evaluated through the calculation of the von Mises stress contact pressure and displacement.

**Results:**

Compared to conventional CMNs, the PFBN group demonstrated a 9.36%–59.32% reduction in the maximum VMS at the implant. The A3.3 ROI fracture (75% bone density) was the most unstable type of fracture. In comparison to conventional CMNs, PFBN demonstrated more stable data, including VMS values (implant: 506.33 MPa, proximal fracture fragment: 34.41 MPa), contact pressure (13.28 MPa), and displacement (17.59 mm).

**Conclusion:**

Compared to the PFNA, ITN, and GN, the PFBN exhibits improvements in stress concentration, stress conduction, and overall model stability in ROI fractures. The double triangle structure aligns better with the tissue structure and biomechanical properties of the proximal femur. Consequently, the PFBN has significant potential as a new fixation strategy for the clinical treatment of ROI fractures.

## Introduction

Reverse obliquity intertrochanteric (ROI) fractures, categorized as OTA/AO 31-A3, represent 2%–23% of trochanteric hip fractures (Haidukewych et al., 2001; Makki et al., 2015; Sekimura et al., 2023). These fractures, identified by a fracture line running from distal-lateral to proximal-medial, present a substantial orthopedic challenge owing to their unique anatomical and mechanical characteristics (Haidukewych et al., 2001). Common in the elderly, these fractures are inherently unstable and often coexist with conditions like osteoporosis, heightening the risks of post-operative complications and functional limitations. The atypical fracture pattern, running perpendicular to the usual inferomedial trajectory from greater to lesser trochanter, often impedes the effectiveness of conventional fixation methods, such as the sliding hip screw (Kyriakopoulos et al., 2022). Conventional implants designed for intertrochanteric fractures struggle to accommodate the specific biomechanical demands of reverse obliquity fractures, leading to issues such as excessive medialization and potential distraction.

The management of ROI fractures is a subject of ongoing debate; however, an expanding body of evidence supports the use of cephalomedullary nail (CMN) fixation. The Proximal Femoral Nail Antirotation (PFNA), interTan nail (ITN), and Gamma nail (GN) have emerged as primary treatments for ROI fractures, each offering distinct biomechanical attributes (Grønhaug et al., 2023). Nonetheless, the limited effectiveness of these CMNs in addressing ROI fractures arises from concurrent damage to the femur’s medial and lateral walls. Studies indicate significant rates of implant-related complications associated with CMNs, including femoral shaft fractures (ranging from 11% to 17%), fixation failure (ranging from 3% to 27%), and reported complications related to distal locking (at 10%) (Schipper et al., 2004; Chou et al., 2012). Of particular concern for mechanical stability, post-operative complications such as screw cut-out, varus collapse, and inadequate rotational stability present significant risks. Any postoperative reoperation in elderly patients suffering from ROI fractures is serious, exposing the patient to additional reoperations, prolonged recovery, and increased mortality. Despite extensive literature on risk factors, the array of choices and available implants, and recent developments such as trochanteric locking plates, antirotational screws, and cement-augmented fixation techniques, the issue of fixation failure remains inadequately addressed (Kouzelis et al., 2014; Neuerburg et al., 2016; Ehlinger et al., 2020). It is thus paramount to minimize the risk of reoperation following treatment of ROI fractures.

The postoperative complications of ROI fracture are higher, which is closely associated with the instability in the proximal region of CMNs. In addressing challenges associated with internal fixation, Zhang Y et al. proposed that a triangular support theory could reduce the risk of failure in fixing intertrochanteric fractures (Zhao et al., 2023). The Proximal Femoral Bionic Nail (PFBN) has been developed with design enhancements aimed at improving biomechanical compatibility with the proximal femur. Notable advancements comprise a reconstruction fulcrum mirroring the normal anatomical fulcrum, a flat outer-side design reducing extrusion, and a unique double triangle structure offering heightened stability.

In spite of reported clinical successes, the existing literature frequently lacks comprehensive comparative data that could elucidate the relative efficacy of various nailing systems. Moreover, previous studies comparing outcomes of different nail designs are limited in scope and seldom involve the comparison of more than two distinct designs (Kwak et al., 2022; Linhart et al., 2023; Mao et al., 2023). In this finite element analysis, we employed four intramedullary fixation approaches—PFBN, Proximal Femoral Nail Antirotation (PFNA), interTan nail (ITN), and Gamma nail (GN)—to address three variations of ROI fractures (AO/OTA 31-A3). Our assessment of the biomechanical performances of these diverse fixation techniques included the computation of implant strength, fixation stability, and the contact pressure of fracture surfaces in normal and osteoporotic property models.

The objective of this finite element analysis was to compare the biomechanical stability between the PFBN and existing CMNs in treating ROI fractures. We hypothesize that the PFBN, with its unique design, will offer superior fixation and stability for this specific fracture type, providing a theoretical basis for its clinical application.

## Materials and methods

Ethical approval for this study (NO. KS 2022-011-1) was provided by the Medical Ethics Committee of the Hebei Medical University Third Hospital.

### Three-dimensional modeling

A formalin-preserved femur from a deceased male individual (age at death: 50 years) was chosen. Prior to further analysis, X-ray examination was conducted to exclude bone abnormalities such as severe osteoporosis, deformities, and tumors. Subsequently, the femur underwent computed tomography scanning (SOMATOM Definition AS scanner, Siemens, Germany, thickness, 0.625 mm; resolution, 512 × 512 pixels). The DICOM format data was imported into Mimics 20.0 software (Materialise, Leuven, Belgium) for geometric model reconstruction and exported in STL format. The STL file was imported into Geomagic Studio 13.0 software (Geomagic Company, United States), and solidification was achieved through operations such as denoising, smoothing, and surface fitting.

### Establishing implants and assembled model

**FIGURE 1:**
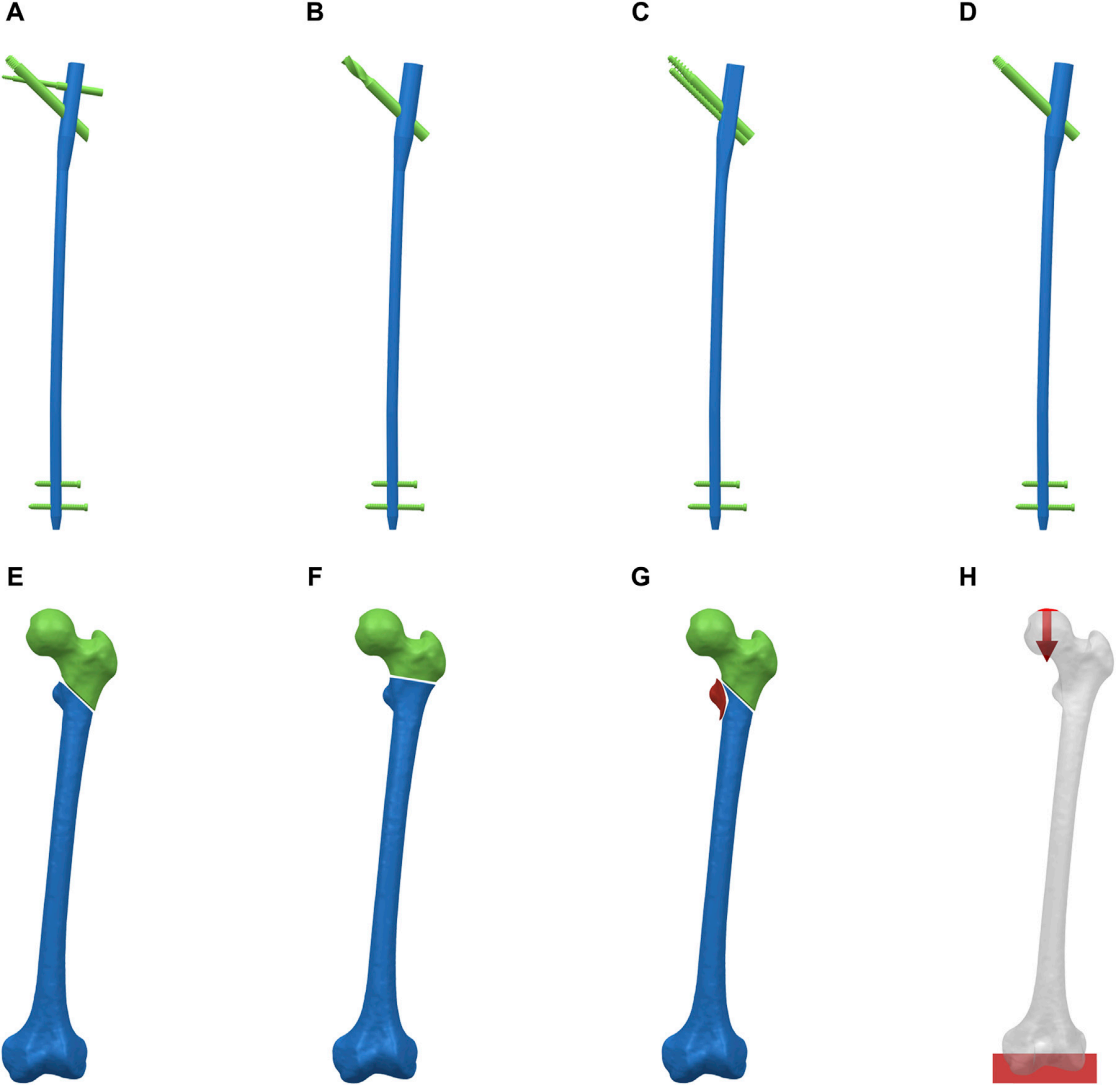
Reverse obliquity intertrochanteric fracture models and cephalomedullary nail models. **(A)** PFBN. **(B)** PFNA. **(C)** ITN, **(D)** GN. **(E)** AO/OTA 31-A3.1 fracture. **(F)** AO/OTA 31-A3.2 fracture. **(G)** AO/OTA 31-A3.3 fracture. **(H)** Boundary and loading conditions.

Implant design and fracture model construction were performed using NX 1980 software (Siemens Company, United States). Four CMN models (PFBN, PFNA, ITN, and GN) were designed based on the implant data parameters provided by Double Medical Technology Inc (China) (Figures 1A–D). ROI fractures geometries were designed according to previously published results of 3D fracture mapping and the 2018 OTA/AO Fracture and Dislocation Classification Compendium (Figures 1E–G) (Meinberg et al., 2018; Li et al., 2019). Then the implants were assembled on the ROI fractures models. All CMNs had consistent diameter (10 mm), angle (125°), and length (400 mm). Furthermore, to maintain uniformity in nail positioning across groups, the nails were positioned identically in the intermediate location within the femoral canal for all groups. All CMNs were centrally placed within the femoral heads, ensuring a standardized tip-apex distance (TAD) of 20 mm. The solid models were meshed with C3D4 elements using Hypermesh 2014 software (Altair Company, United States).

### Material properties and boundary conditions

Bone density is related to the material properties. Hence, the material properties of each femoral model were based on the Hounsfield units from the CT scan data in Mimics 20.0 software (Jiang et al., 2023). The mathematical formulas are as follows, where *ρ* was the bone density, HU represented the Hounsfield units, *E* was Young’s modulus, and *ν* was Poisson’s ratio: (1)*ρ*(g/cm^3^) = 0.000968*HU+0.5, (2) If *ρ* ≤ 1.2 g/cm^3^; *E* = 2014*ρ* ^2.5^(MPa), *ν* = 0.2, (3) If *ρ* > 1.2 g/cm^3^; *E* = 1763*ρ* ^3.2^(MPa), *ν* = 0.32. To simulate models with osteoporosis, bone density was reduced to 75% of the initial bone density, following a previously established study protocol (Goffin et al., 2013). The models were imported into Abaqus 6.14 (Dassault company, United States).

All the implants were assigned as titanium alloy (Ti-6Al-4V), with *E* of 110,000 MPa and *v* of 0.3. All nodes at the distal femur were mechanically fixed by constraining 6 degrees of freedom. All contact types were defined according to the Coulomb friction law: bone–bone (friction coefficient: *μ* = 0.46), bone–implant (*μ* = 0.3), and implant–implant (*μ* = 0.2) (Xia et al., 2021; Yang et al., 2023). The screw thread was bonded to the bone (Zhang et al., 2023).

The femur model was abducted by 10° and tilted backward by 9°. A single-cycle load condition of 2100 N was applied to the finite element models using distributed coupling conditions, ensuring the uniform distribution of individual force over the bone tissue surface corresponding to the area of the femoral head (Figure 1H) (Xia et al., 2021). The direction was normal standing angle vertical down, and the distal end of the femur was completely fixed.

### Verification of finite element models

The von Mises stress (VMS) on the intact femur was tested to analyze the mesh convergence. The convergence criterion used was a change of <5% (Zhang et al., 2023). The mesh size was set to 2 mm. The implant components were meshed with 1.0 mm, which was fine enough to preserve geometrical features (Zeng et al., 2020). The mean element numbers of the four groups were 672,555 (PFBN), 698,487 (PFNA), 735,814 (ITN), 662,348 (GN).

**FIGURE 2:**
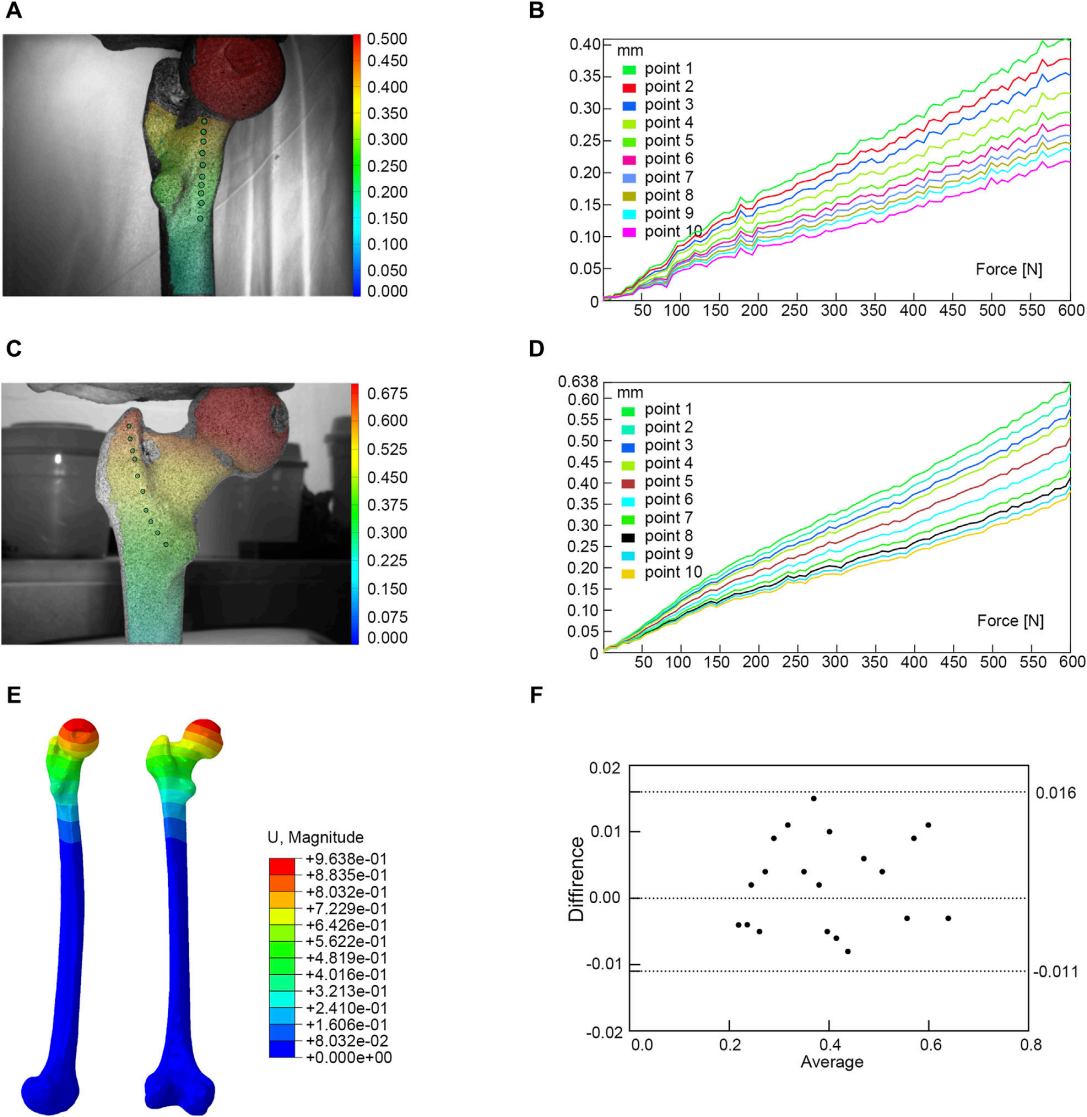
Finite element models validation. **(A)** Anterior view of biomechanical verification. **(B)** The force-displacement curve of anterior marker points. **(C)** Posterior view of biomechanical verification. **(D)** The force-displacement curve of posterior marker points. **(E)** Finite element model validation. **(F)** Bland-Altman analysis between biomechanical study and finite element analysis.

We utilized the same femur for mutual validation of biomechanical and finite element models. The BOSE ElectroForce 3520-AT (BOSE Company, United States) was employed to apply axial pressure ranging from 0 to 600 N onto the surface of the femoral head at a rate of 5 N/s. Concurrently, the high-speed camera integrated within the GOM non-contact optical strain measurement system (GOM GmbH, Germany) captured the loading process at a frame rate of seven frames/s. The resultant images were subsequently subjected to computer processing to derive displacement images and quantify the displacement values specific to the femur under an axial pressure of 600 N. Subsequent to data acquisition, the GOM Software 2021 was employed to select the appropriate starting point for calculations based on the collected images and to define the calculation area. In the software interface, the stability-loaded cloud diagram was selected, and the displacement values corresponding to the chosen points on the femur were quantified (Figures 2A,C). Upon completion of the calculations, the force-displacement curves were automatically generated (Figures 2B,D; Table 1).

**TABLE 1:** The displacement values of marker points in biomechanical and finite element study.

Point		1	2	3	4	5	6	7	8	9	10
Finite element analysis (mm)	anterior	0.396	0.362	0.348	0.312	0.285	0.270	0.263	0.243	0.238	0.221
	posterior	0.641	0.594	0.566	0.558	0.505	0.467	0.447	0.418	0.399	0.379
Biomechanical study (mm)	anterior	0.406	0.377	0.352	0.323	0.294	0.274	0.258	0.245	0.234	0.217
	posterior	0.638	0.605	0.575	0.555	0.509	0.473	0.434	0.412	0.394	0.381

Under the same loading and boundary conditions as the biomechanical experiment, the displacement values at the corresponding position were calculated for the normal femur finite element model (Figure 2E; Table 1). Bland-Altman analysis was employed to assess the agreement between the biomechanical study and finite element analysis using GraphPad Prism 8.0 statistical software.

### Evaluation indices

The VMS and displacement of the implant models were calculated to evaluate the biomechanical stability. The stability of the proximal fracture fragment was assessed through VMS of the proximal femur and contact pressure on the fracture surface (Cheng et al., 2023; Zhan et al., 2024).

## Results

### Finite element model verification result

The result of the Bland-Altman analysis indicated that there was good agreement between the results of the biomechanical study and the finite element analysis, suggesting that our model was appropriate for the subsequent study (Figure 2F).

### The VMS distributions of the implant models

**FIGURE 3:**
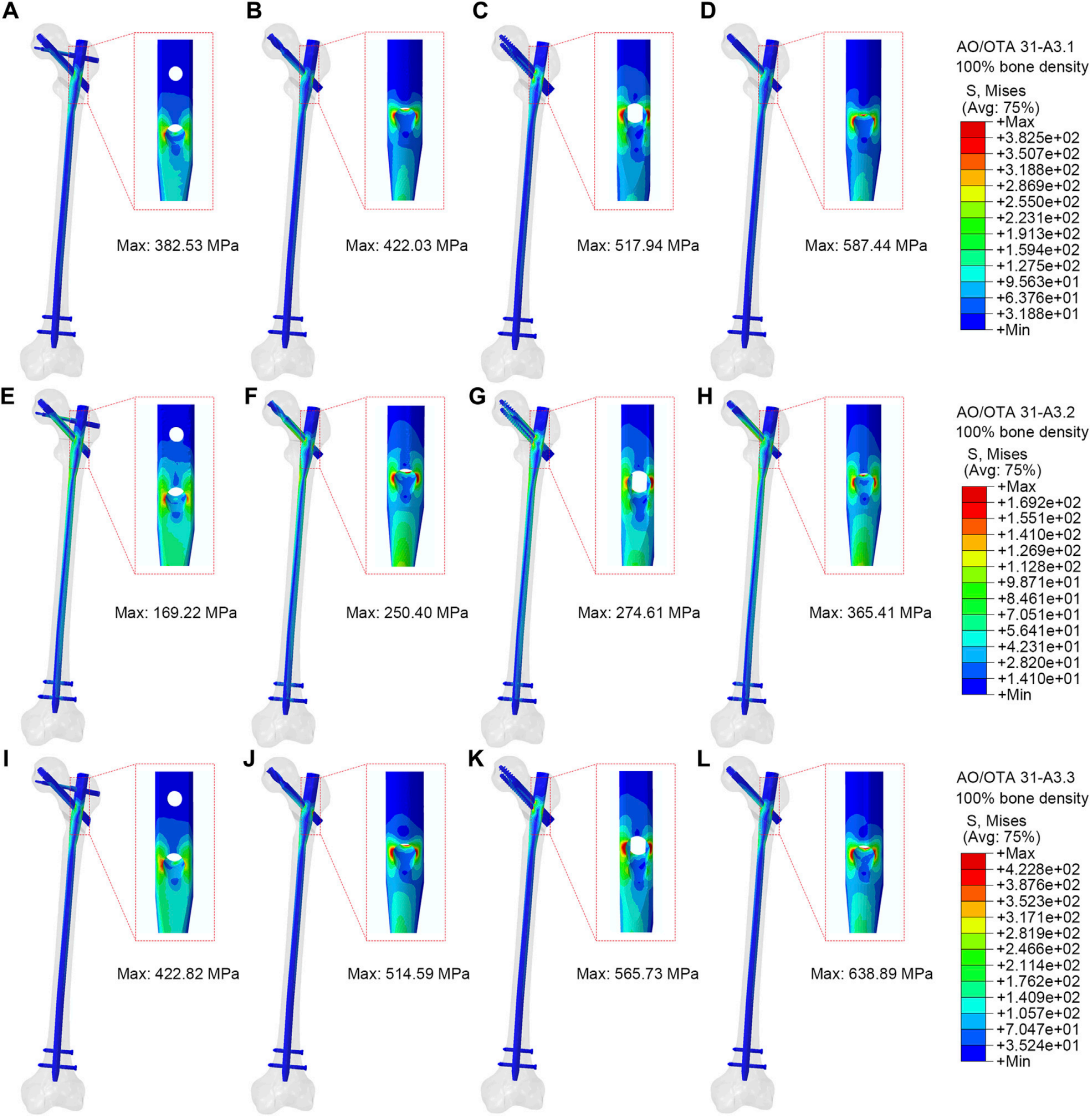
Stress distribution of different implants (100% bone density). **(A,E,I)** were PFBN of A3.1, A3.2 and A3.3 ROI fracture. **(B,F,J)** were PFNA of A3.1, A3.2 and A3.3 ROI fracture. **(C,G,K)** were ITN of A3.1, A3.2 and A3.3 ROI fracture. **(D,H,L)** were GN of A3.1, A3.2 and A3.1, A3.2 and A3.3 ROI fracture.

Figure 3 showed stress on implants for four fixation models. In the A3.1 ROI fracture group, the peak VMS values for PFBN, PFNA, ITN, and GN are 382.53 MPa, 422.03 MPa, 517.94 MPa, and 587.44 MPa, respectively (Figures 3A–D). Similarly, in the A3.2 and A3.3 fracture groups, the trend is consistent: PFBN consistently shows the smallest peak VMS, followed by PFNA, ITN, and GN, indicating that PFBN provides the most stability in both A3.2 and A3.3 fracture scenarios (Figure 3E-L). Except for the A3.2-type ROI fracture group, where the maximum stress of ITN was located between the integrated interlocking screw, the maximum stress was recorded at the intersection of the fixating screw and the main nail for all other models. In the osteoporosis models (75% bone density), implants stress significantly increases (Supplementary Figure S1; Table 2). Taking the A3.3 fracture model as an example, PFBN, PFNA, ITN, GN were 1.20, 1.31, 1.23, and 1.50 times that of the normal bone model (100% bone density), respectively. PFBN still exhibited the smallest VMS (506.33 MPa), reducing by 25.15%, 27.16%, 47.04% compared to PFNA, ITN, GN, respectively. The GN group had the highest VMS in all fracture type, and reached the yield strength of titanium alloy in the (75% bone density) model, suggesting that GN was not suitable for application in the A3.3 fracture type. The peak values and distributions of VMS for each CMNs were depicted in Figure 3. This suggests that PFBN optimizes the distribution of implant stress, avoids stress concentration, and enhances the mechanical stability and strength.

**TABLE 2:** The von Mises stress distribution and displacement of osteoporosis finite element model (75% bone density).

	PFBN			PFNA			ITN			GN		
	A3.1	A3.2	A3.3	A3.1	A3.2	A3.3	A3.1	A3.2	A3.3	A3.1	A3.2	A3.3
VMS of PFF (MPa)	25.11	30.37	34.41	31.42	31.57	36.70	34.90	38.45	39.34	34.45	36.93	38.48
VMS of implant (MPa)	487.18	247.42	506.33	593.41	455.28	676.44	622.16	510.87	695.14	729.51	608.20	956.15
displacement (mm)	16.90	17.03	17.59	17.35	17.18	17.63	17.74	17.55	18.23	17.83	17.80	18.14
contact pressure (MPa)	9.18	20.92	13.28	9.96	22.70	16.98	10.41	29.63	17.57	14.81	23.46	17.49

PFBN, proximal femoral bionic nail; PFNA, proximal femoral nail antirotation; ITN, InterTan Nail, GN, gamma nail; VMS, von Mises stress; PFF, proximal fracture fragment.

### The displacement distributions of the models

**FIGURE 4:**
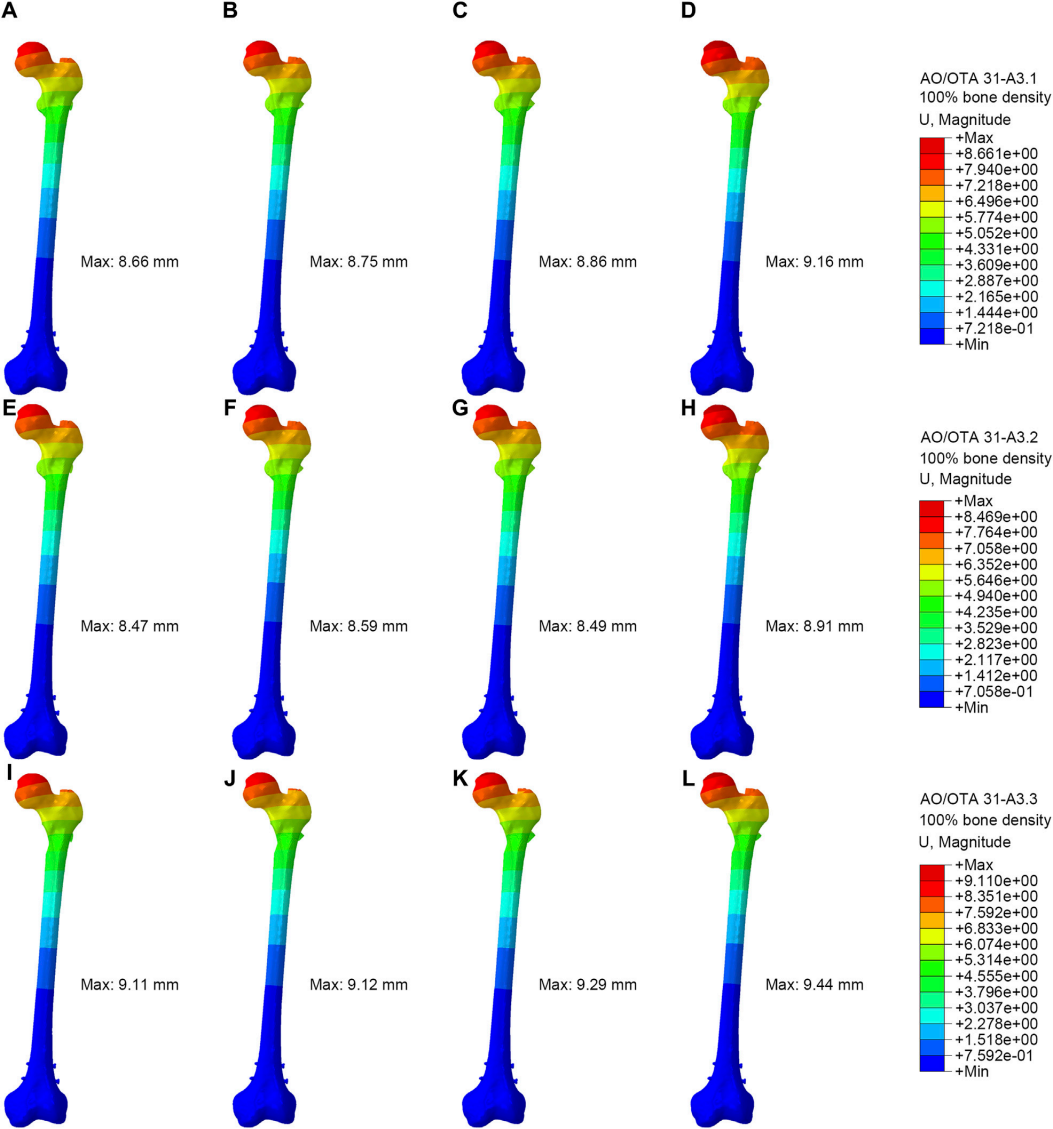
Displacement distribution of the femur and implants (100% bone density). **(A,E,I)** were PFBN of A3.1, A3.2 and A3.3 ROI fracture. **(B,F,J)** were PFNA of A3.1, A3.2 and A3.3 ROI fracture. **(C,G,K)** were ITN of A3.1, A3.2 and A3.3 ROI fracture. **(D,H,L)** were GN of A3.1, A3.2 and A3.3 ROI fracture.

Figure 4 showed the displacement distribution in four models with different types RIO fractures. The maximum displacements were located at the top of the femoral head for all models. The PFBN showed smallest maximum displacement compared with conventional CMNs. The stability of the A3.3 ROI fracture was relatively poor, with the maximum displacement. In the 100% bone density model, the maximum displacements for PFBN, PFNA, ITN, and GN were 9.11 mm, 9.12 mm, 9.29 mm, and 9.44 mm, respectively. In the 75% bone density models, when axial loads of 2100 N were applied, the maximal displacements for PFBN, PFNA, ITN, and GN were 17.59 mm, 17.63 mm, 18.23 mm, and 18.14 mm (Supplementary Figure S2; Table 2).

### The VMS distributions of the proximal fracture fragment

**FIGURE 5:**
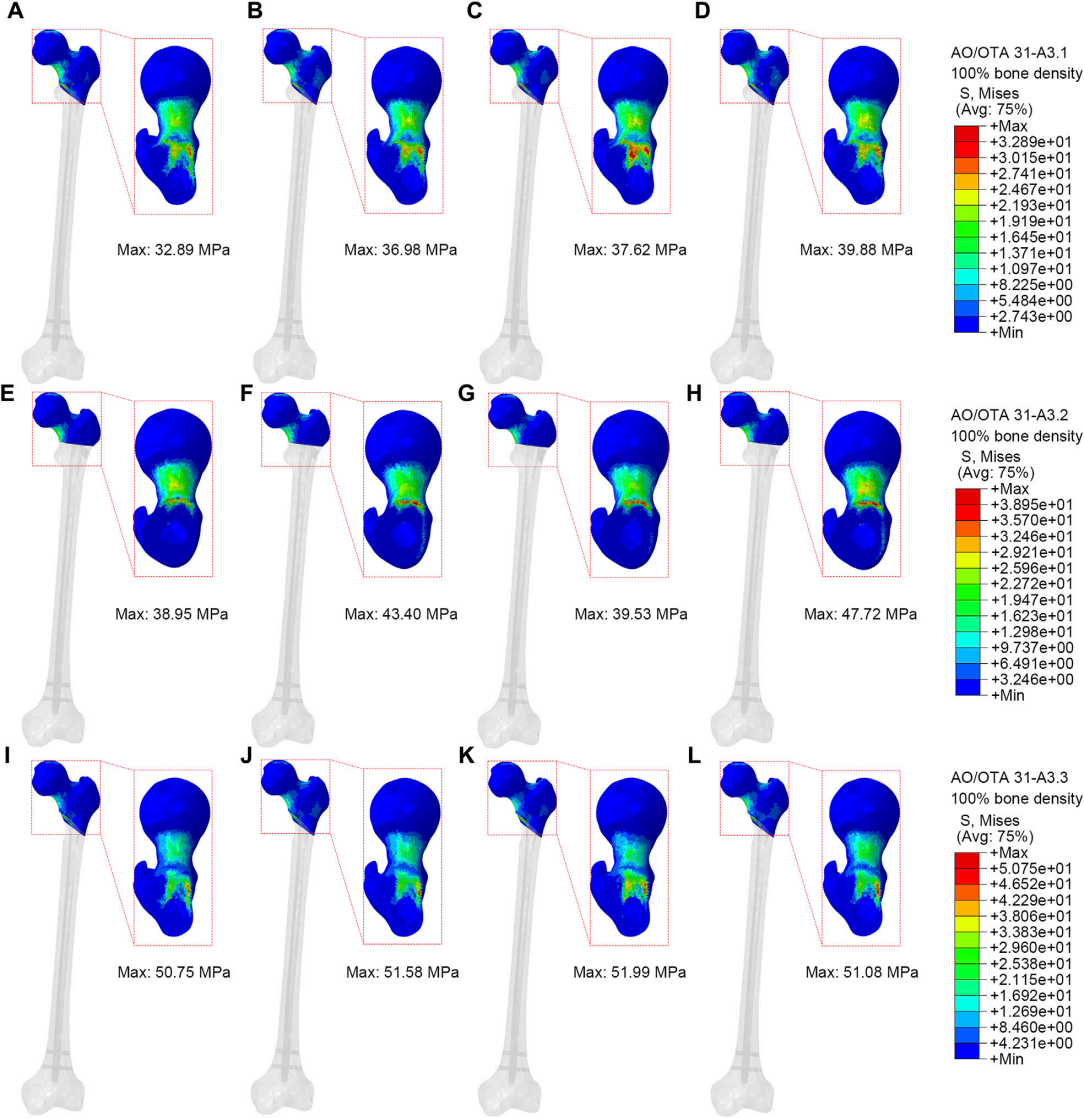
Stress distribution of proximal fracture fragment (100% bone density). **(A,E,I)** were PFBN of A3.1, A3.2 and A3.3 ROI fracture. **(B,F,J)** were PFNA of A3.1, A3.2 and A3.3 ROI fracture. **(C,G,K)** were ITN of A3.1, A3.2 and A3.3 ROI fracture. **(D,H,L)** were GN of A3.1, A3.2 and A3.3 ROI fracture.

Figure 5 showed VMS of proximal fracture fragment of the femur under axial loads of 2100 N. In the A3.1 ROI fracture group, the peak VMS at the proximal fracture fragment of the femur for PFBN, PFNA, ITN, and GN was 32.89 MPa, 36.98 MPa, 37.62 MPa, and 39.88 MPa, respectively. Similarly, in the A3.2 fracture group, the corresponding peak VMS values for PFBN, PFNA, ITN, and GN at the proximal fracture fragment are 38.95 MPa, 43.40 MPa, 39.53 MPa, and 47.72 MPa. Likewise, in the A3.3 ROI fracture group, the peak VMS at the proximal fracture fragment of the femur for PFBN, PFNA, ITN, and GN is 50.75 MPa, 51.58 MPa, 51.99 MPa, and 51.08 MPa. The VMS trend of the osteoporosis model at the proximal fracture fragment aligns with the normal model (Supplementary Figure S3; Table 2). Using the A3.3 fracture model as an example, the peak VMS at the proximal fracture fragment of PFBN decreased by 6.24%, 12.53%, and 10.58% compared to the PFNA, ITN, and GN groups, respectively. Lower stress values indicate that the internal fixation devices bear the stress at the proximal end of the femur, thereby avoiding stress concentration and the occurrence of complications such as cut-out.

### Contact pressure of fractured surfaces

**FIGURE 6:**
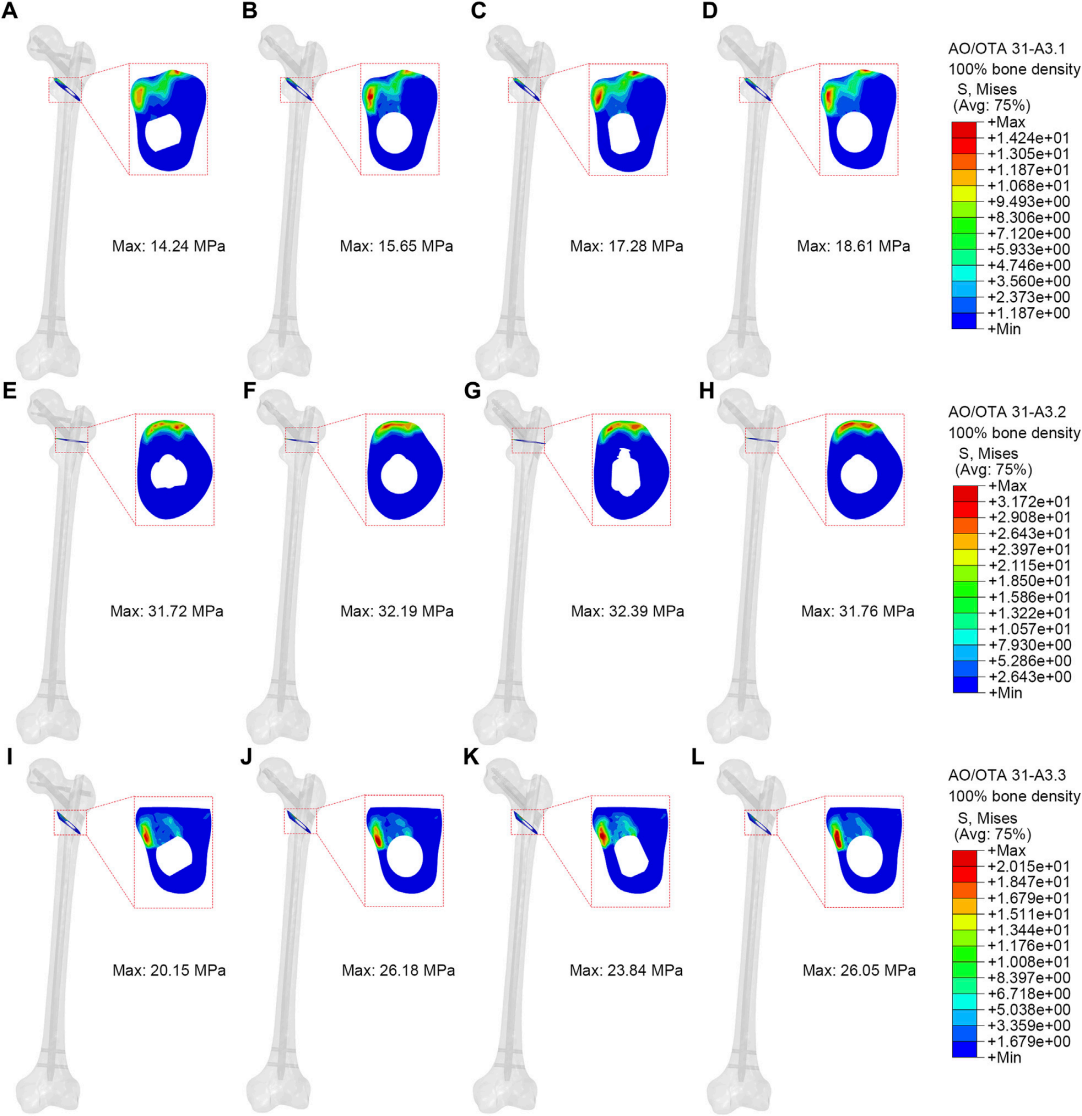
Contact pressure of fractured surface (100% bone density). **(A,E,I)** were PFBN of A3.1, A3.2 and A3.3 ROI fracture. **(B,F,J)** were PFNA of A3.1, A3.2 and A3.3 ROI fracture. **(C,G,K)** were ITN of A3.1, A3.2 and A3.3 ROI fracture. **(D,H,L)** were GN of A3.1, A3.2 and A3.3 ROI fracture.

Higher contact pressure on fractured surface indicates higher stress at the fracture site, which may impact the stability of the fracture. Figure 6 illustrated the contact pressure at the fracture surface of the models. The end contact pressure of the PFBN group for A3.1, A3.2, and A3.3 fracture types (100% bone density) were 14.24 MPa, 31.72 MPa, and 20.15 MPa, respectively. The end contact pressure of the PFBN group for A3.1, A3.2, and A3.3 fracture types (75% bone density) were 9.18 MPa, 20.92 MPa, and 13.28 MPa, respectively (Supplementary Figure S4; Table 2). The PFBN group had the lowest end contact pressure among all fracture types and bone densities, indicating that the PFBN group had the best stability and avoided postoperative mechanical complications such as screw cut-out and varus collapse.

**FIGURE 7:**
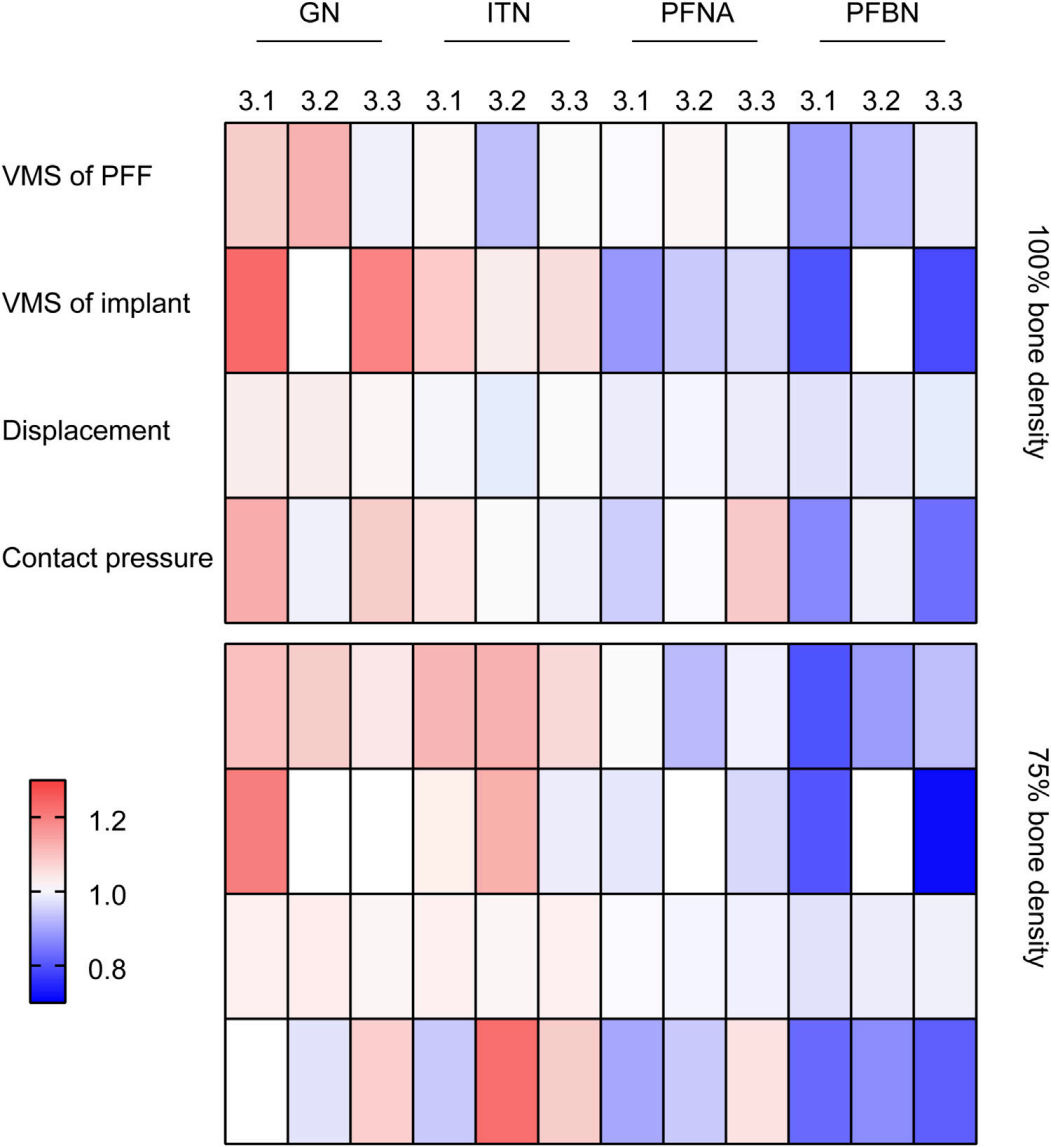
Heatmap of normalization data for each group.

The present study conducted normalization of the data for each group (X/‾X), and the results were presented using a heatmap (Figure 7). It can be concluded that PFBN outperforms the conventional CMN group in terms of implant stress, displacement of whole model, proximal fracture fragment stress, and contact pressure on fractured surface.

## Discussion

In this finite element study, we compared the biomechanical stability of PFBN and conventional CMNs, namely, PFNA, ITN, and GN, in treating ROI fractures with varying bone densities (100% and 75%). Compared to conventional CMNs, the PFBN group demonstrated a 9.36%–59.32% reduction in the maximum VMS at the implant. The A3.3 ROI fracture (75% bone density) was highly unstable, whereas PFBN showed superior stability in VMS values (implant: 506.33 MPa, proximal fracture fragment: 34.41 MPa), contact pressure (13.28 MPa), and displacement (17.59 mm). Underpinned by Zhang’s N triangle theory (Ding et al., 2023), our study demonstrates that, during the fixation of ROI fractures, PFBN optimizes the stress distribution between the implant and the proximal fracture fragment of the femur. The inclusion of the supporting screw effectively diminishes stress concentration in the fixating screw, thereby contributing to the mitigation of potential risks associated with withdrawal, cut-out, and hip varus.

The use of intramedullary nails has become the preferred approach for addressing intertrochanteric fractures, particularly those associated with osteoporosis. However, the efficacy of conventional CMNs in managing ROI fractures may be suboptimal, particularly when both the medial and lateral walls of the proximal femur are compromised simultaneously. In a study by Irgit et al., AO/OTA 31-A3 type fractures treated with intramedullary nailing showed a 0.6% intraoperative complication rate. Postoperatively, complications occurred in 12% of cases, leading to reoperations in 8%. The 1-year mortality rate was determined to be 10.1% (Irgit et al., 2015). For elderly patients with hip fractures, the additional surgeries triggered by these complications can lead to significant functional impairment, necessitating surgical interventions such as revision procedures or arthroplasty.

Early surgical stabilization of fractures is crucial for minimizing complications related to prolonged immobilization and reducing the risk of mortality. Conventional CMNs primarily undergo evolution at the fixating screws, involving an increase in screw quantity and structural changes to the screw heads. These adaptations confer good resistance to compressive forces resulting from medial wall fractures. However, when it comes to lateral wall damage in reverse oblique fractures, known as the tension side, the design philosophy of conventional CMNs lacks the capability to withstand tension forces at the proximal femur. Consequently, there is a higher incidence of internal fixation failure in such cases.

The most common complication resulting from proximal instability in conventional CMNs is the “Z-effect”. The dual-screw design of reconstruction nails provides a certain level of rotational control; nevertheless, it may give rise to the “Z-effect” phenomenon, marked by lateral migration of the lag screw and medial migration of the superior lag screw during loading. The PFNA is also susceptible to the “Z-effect”, with literature reporting an incidence ranging from 5% to 10% with PFNA nails (Parry et al., 2018; Ehlinger et al., 2020). In contrast, the design of the ITN effectively alleviates the “Z-effect” phenomenon; however, it comes at the cost of a reduction in the bone volume surrounding the femoral calcar, thereby resulting in decreased stability at the proximal end of the femur. In addition, the primary shaft of most CMNs, characterized by its cylindrical profile, leading to the occurrence of the “V-effect” on the lateral wall and the head-neck fragments. This iatrogenic “V-effect” can result in damage to the lateral wall, loss of reduction, and varus displacement of the head–neck fragment. Such complications contribute to a decrease in stability and an elevated risk of cut-out (Nie et al., 2020). The decrease in proximal femoral stability is manifested as an increase in displacement and contact pressure in this finite element study.

Moreover, Ward’s Triangle plays a crucial role in the biomechanics of the hip joint, particularly in relation to hip fractures. The ROI fractures involve the posteromedial or lateral bone quality of the subtrochanteric region, it will damage the structural integrity of Ward’s Triangle, further aggravating the fracture instability and implant sliding or cut-out. Therefore, the ideal CMNs for stabilizing ROI fractures would be an implant capable of reconstructing Ward’s Triangle to counteract the lateral cortical tension, Z-effect, V-effect, and varus angulation of the proximal fragment.

In contrast to conventional CMNs, the innovation of PFBN resides in its double triangle structure, which includes the main screw, fixating screw, and supporting screw. The first angle, called mixed triangle, was made of cancellous bone, fixating screw and supporting screw in femoral head. The second angle, called metal triangle, was composed of the main screw, fixating screw, and supporting screw. The double triangle structure enhances the hardware rotational strength in three-dimensional space and forms a robust mechanical relationship with the fragments of proximal femoral fractures. As our result showed, compared to conventional PFNA, ITN, and GN, the PFBN group demonstrated a 3.8%–28.05% reduction in the maximum VMS at the proximal femur (75% bone density). Zhang et al. developed an equivalent biomechanical model to analyze the impact of PFBN on the bone reconstruction of unstable intertrochanteric femur fractures. The results are consistent with our findings, indicating that, compared with PFNA and InterTan, PFBN can significantly reduce the maximum strain in the proximal femur (Zhang et al., 2024). The cross structure of the supporting screw and fixating screw establishes a new pivot point. In contrast to the relatively longer lever arm on the weight-bearing side of conventional CMNs, the internal lever arm of the new pivot point in PFBN is shorter, while the external lever arm is longer. The reconstructed new pivot point restores the physiological support points formed by trabecular bone beams under pressure and tension conditions at the proximal femur, thereby reducing stress concentration and enhancing stability. Moreover, the supporting screw and the main nail are in a locking relationship, significantly sharing the tension generated by the fixating screw on the lateral cortical bone of the femur. Consequently, in this study, due to the new reconstructed pivot point, PFBN exhibits a 9.36%–59.32% reduction in implant stress.

In the context of A3.3 ROI fractures, the absence of the calcar results in an imbalance of force loading on the femoral head, leading to the generation of a moment on the proximal fragment. Consequently, the proximal fragment exhibits a tendency to rotate into a varus position. The implant serves as the only supportive mechanism capable of effective support. The greater the stress value is, the more likely the screw cut-out will occur. Makki et al. reported a 22.2% failure rate of PFNA in 58 patients with ROI fractures during postoperative follow-up (Makki et al., 2015). Vaquero et al. compared the treatment of unstable intertrochanteric fractures with PFNA and GN, finding similar complication rates of 45% and 40%, respectively (Vaquero et al., 2012). In the A3.3 ROI osteoporotic fracture model of this study, the PFNA implant stress was 676.44 MPa, ITN was 695.14 MPa, and GN was 956.15 MPa, respectively. Compared with conventional CMNs, the implant stress in the PFBN group decreased by 17.90%–59.32%. Therefore, the double triangle structure of PFBN reconstructs the damaged Ward’s Triangle, thus enhancing the overall stability of the model and avoiding potential risks associated with withdrawal, cut-out, and hip varus.

Clinical benefits associated with faster healing include an earlier return to function and a reduced risk of fixation failure due to a shorter load-sharing duration of the implant (Bottlang et al., 2016). Proper pressure at the end of hip fracture can promote fracture healing by stimulating these processes and enhancing bone formation. However, excessive pressure will lead to unstable fracture by disrupting these processes and causing bone resorption (Cui et al., 2020). ROI fractures generate shear forces across the fracture site, resulting in medialization and shortening of the shaft with varus angulation and external rotation of the proximal fragment. Therefore, during the early stage of ROI fracture healing, it is important to focus on minimizing movement between the fracture fragments to achieve relative stability. The PFBN group, with its double triangle structure and newly reconstructed pivot point, may partially decrease the contact pressure on the fractured surface compared to conventional CMNs. The present study demonstrated that PFBN decreases contact pressure on the fractured surface by 7.8%–38.04% (75% bone density). The comparison analysis from our finite element model has been also supported by recent clinical studies (Zhao et al., 2023). The 1-year follow-up of 12 patients with unstable intertrochanteric femoral fractures treated by PFBN revealed that the Harris and Parker-Palmer scores were both good.

This study has several limitations. Firstly, the presupposed properties of materials were isotropic, homogeneous and linear elastic, which was just the simplification of the reality. However, using the material assignments by the method of CT Hounsfield unite and grey values partly remedied this flaw. More notably, in this study, the same femur specimen was used to validate the finite element model through the GOM non-contact optical strain measurement system. Secondly, in this finite element study, the utilization of 75% of normal bone density to simulate osteoporotic patients differed from real-world conditions. Thirdly, the single-plane osteotomy model might have limitations, thus rendering natural fracture models a potentially superior choice (Zhan et al., 2024). Lastly, despite the superior biomechanical stability of PFBN compared to conventional CMNs, randomized controlled studies are still needed to validate its clinical outcomes.

## Conclusion

In summary, compared to the PFNA, ITN, and GN, the PFBN exhibits improvements in stress concentration, stress conduction, and overall model stability in ROI fractures. The double triangle structure aligns better with the tissue structure and biomechanical properties of the proximal femur. Improved load distribution is associated with better long-term outcomes, potentially reducing the likelihood of implant-related issues and enhancing the overall success of fracture treatment. Consequently, the PFBN shows significant potential for the clinical treatment of ROI fractures.

## Data Availability

The original contributions presented in the study are included in the article/Supplementary Material, further inquiries can be directed to the corresponding authors.
