# Supplementary figures and images for: Comparative study of a novel proximal femoral bionic nail and three conventional cephalomedullary nails for reverse obliquity intertrochanteric fractures: a finite element analysis
Source: Front Bioeng Biotechnol. 2024 Jun 13;12:1393154. doi: 10.3389/fbioe.2024.1393154 (PMC11208680; doi:10.3389/fbioe.2024.1393154)

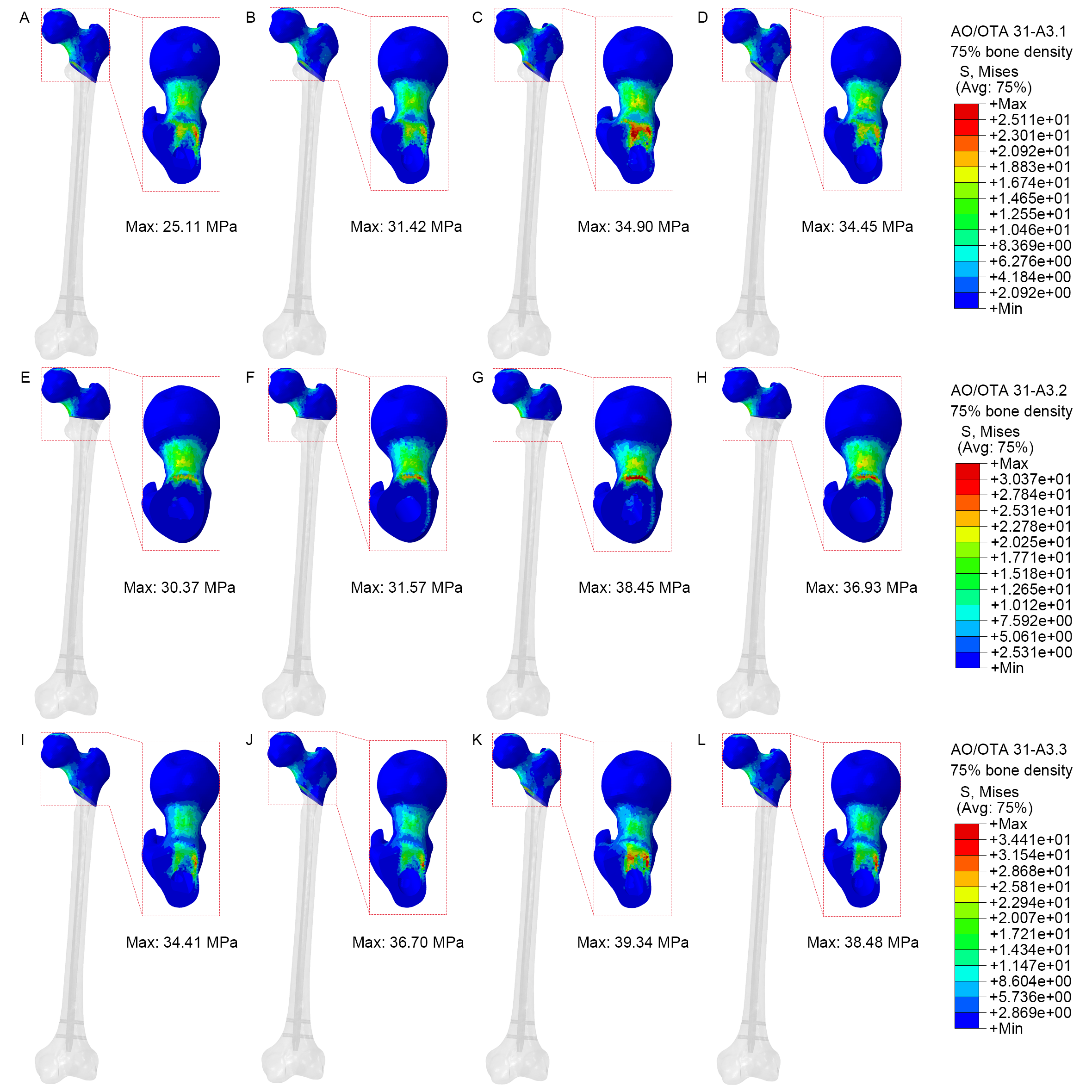

Supplement: Supplementary file 1 [file Image3.TIF]

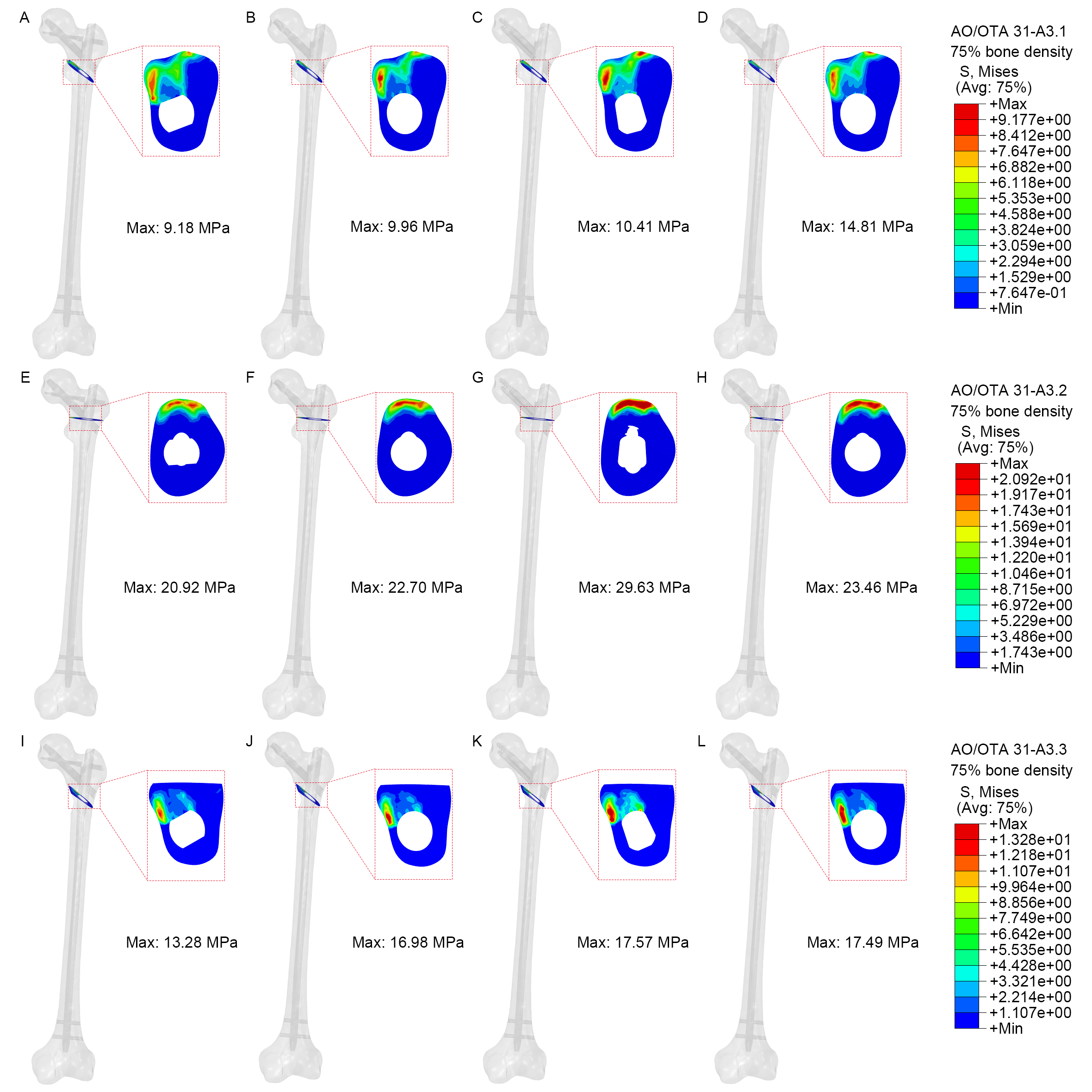

Supplement: Supplementary file 2 [file Image4.TIF]

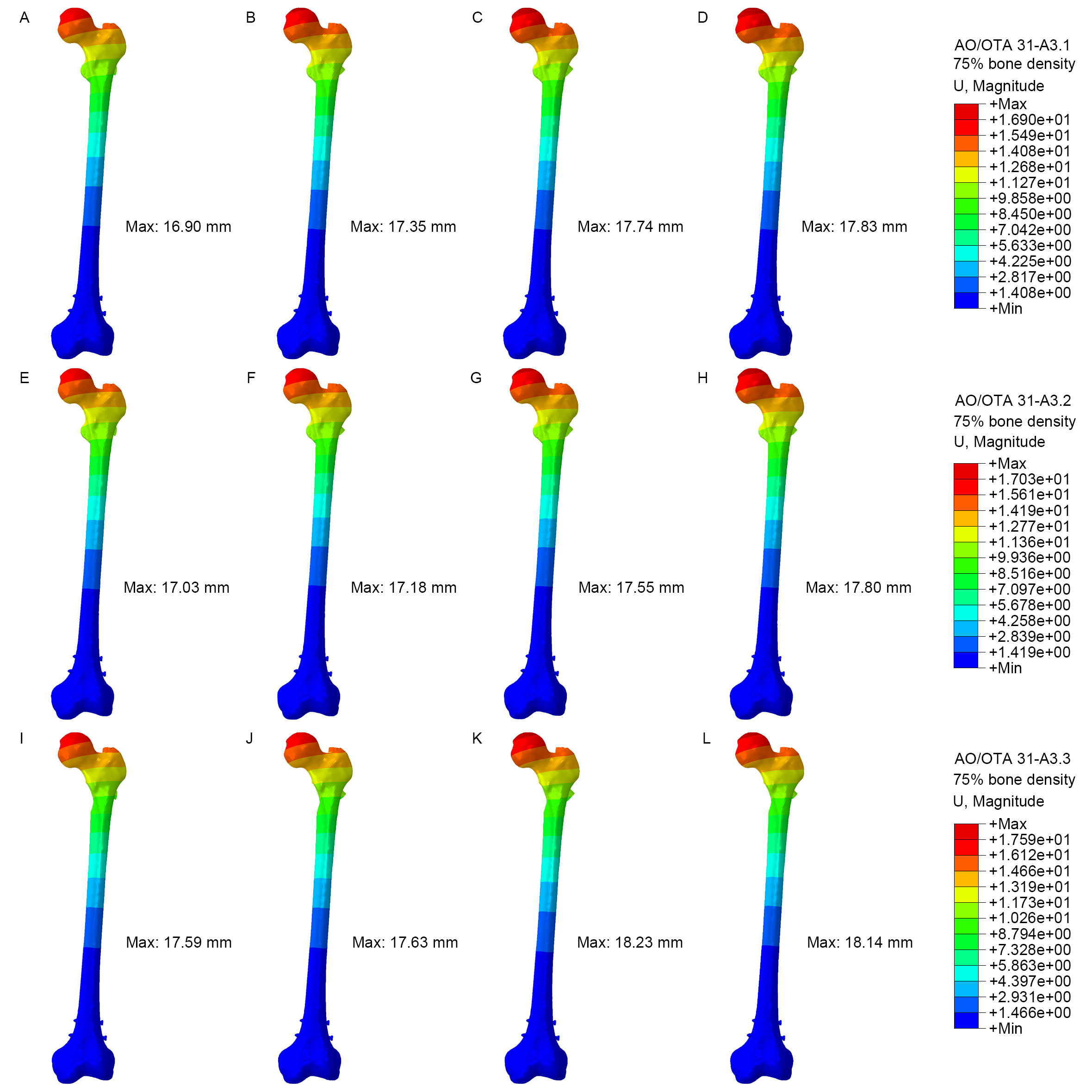

Supplement: Supplementary file 3 [file Image2.TIF]

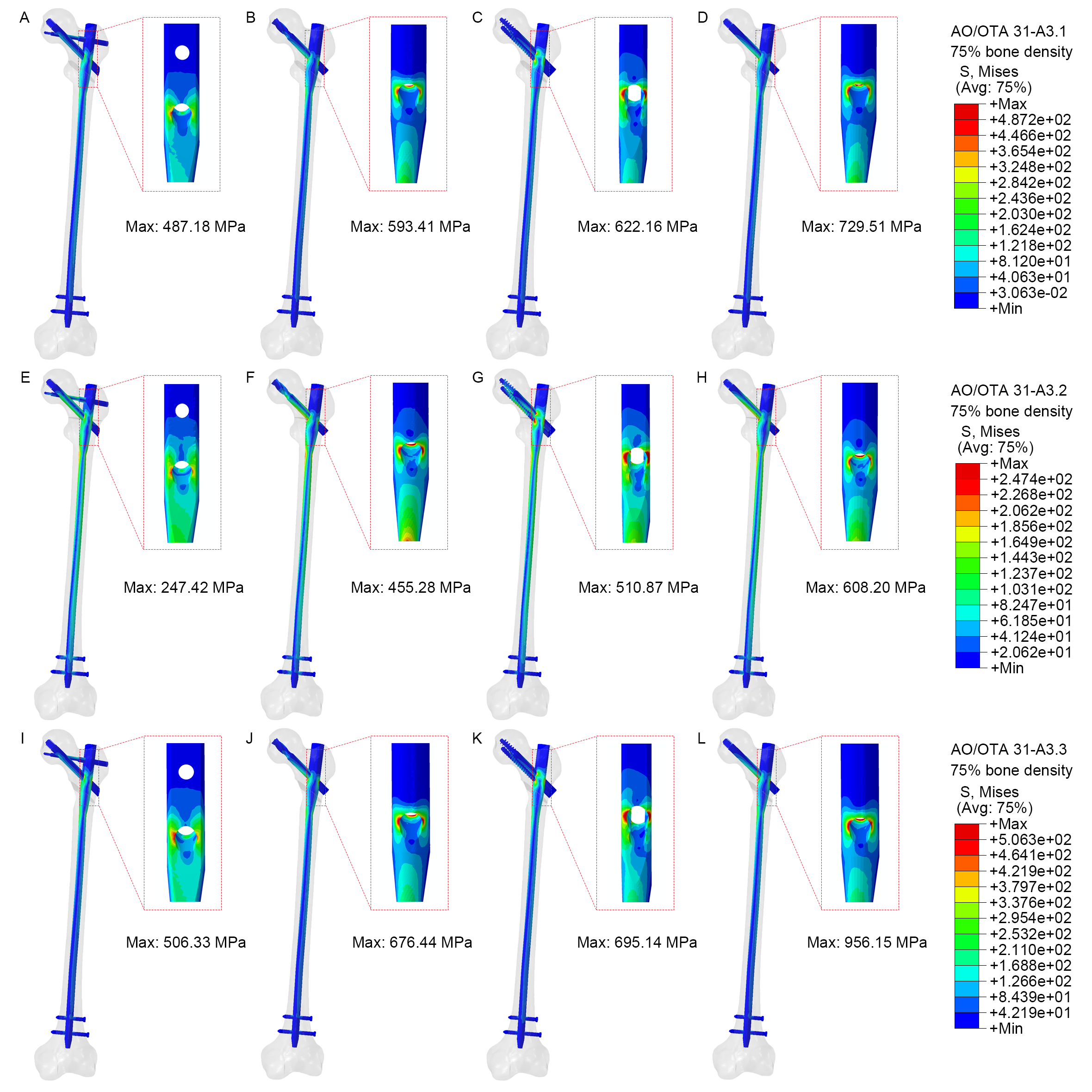

Supplement: Supplementary file 4 [file Image1.TIF]
